# Delivery by Forceps and Subsequent Anasarca

**Published:** 1892-03

**Authors:** John Hall

**Affiliations:** Victoria, B. C.


### DELIVERY BY FORCEPS AND SUBSEQUENT ANASARCA.

(Bureau of Obstetrics, I. H. A. of June, 1891.)

JOHN HALL, VICTORIA, B. C.

The writer was called suddenly one morning, in 1890, to see a woman in labor, which had continued all night, and was thought to be ceasing from exhaustion. On coming here I had given up attending such cases, and with them all the instruments usually required excepting a pair of forceps, but the patient and her husband being old friends of mine, I at once took them in my hand and drove some two miles to his place.

I found the poor woman much exhausted, and almost hopeless, pains being scanty and insufficient. Presentation normal, and parts well dilated, while the face looked very yellow. The use of a remedy came immediately into mind. Unfortunately the nurse and neighbor were violently opposed to anything I

could do in that line, and fearing that my medicine might not speedily act, and that I must in consequence swing from the nearest tree as an impostor, without more ado I at once brought the invalid around in her bed, and warming and oiling the forceps, proceeded to deliver her. The forceps at first slipped, but readjusting and allowing them to *make their own axis*, waiting for pains, soon delivered her of a fine child. This was fortunate for me, as the hostility continued whatever might be done, and other doctors of very doubtful report lived some three miles distant in Victoria.

The patient made a fair recovery for a time, but afterward developed anasarca. Had great tenderness of the right hypochondrium, face yellow and puffed, the upper eyelids being especially swollen. These symptoms, with the difficulty she had long experienced in taking a walk, and soon tiring, had been felt more or less through her pregnancy, indicating a grave hepatic malady. Her symptoms pointed very forcibly to Kali-carb., which was administered in high potencies of the 10M and 19M, Fincke, and CM of Swan, in rare doses, and only when the former seemed to have exhausted its effects, the disease giving way eventually to a great desire for air, which, with other symptoms, called for Pulsatilla. This was accordingly given and completed the cure in about six weeks, she preserving her milk, and making a full recovery, the mother of five healthy children.

